# Assessment of the effects of different frying oils and red beet peel powder on acrylamide formation, lipid oxidation, and quality characteristics of meatballs

**DOI:** 10.3389/fnut.2026.1930556

**Published:** 2026-09-28

**Authors:** Adem Savaş

**Affiliations:** Department of Food Engineering, Hazelnut Specialization Coordination Office, Giresun University, Giresun, Turkey

**Keywords:** acrylamide formation, meat quality, meatball, red beet peel powder, texture profile, vegetable oils

## Abstract

Herein, the effects of red beet peel powder (RBPP) addition at different concentrations (0%, 0.5%, and 1%) and frying in different vegetable oils (sunflower, corn, and hazelnut oils) on the physicochemical properties, color characteristics, lipid oxidation, textural parameters, and acrylamide formation of meatballs were investigated. RBPP concentration significantly affected pH, moisture content, hardness, TBARS, and acrylamide formation (*p* < *0.05*), whereas no significant effects were observed on cooking loss, color characteristics (L^*^, a^*^, b^*^, C^*^, and h°), springiness, cohesiveness, gumminess, chewiness, and resilience. Oil type significantly influenced pH, moisture content, cooking loss, hardness, springiness, gumminess, chewiness, and resilience (*p* < *0.05*), while no significant effects were detected on color characteristics, cohesiveness, TBARS and acrylamide content. Among the RBPP treatments, the highest acrylamide content was determined in meatballs supplemented with 0.5% RBPP, whereas the 1% RBPP treatment produced acrylamide levels statistically comparable to those of the control group. These findings suggest that, in terms of acrylamide levels, the addition of 1% RBPP to meatball formulations may be a more suitable incorporation level than 0.5%.

## 1 Introduction

Recent studies have focused on the reintegration of food processing waste into the economy to both mitigate environmental pollution and generate economic added value (1, 2). Although by-products generated in the fruit and vegetable processing industries are generally discarded as waste, they are actually rich sources of bioactive components, fibers, minerals, and antioxidants. In this context, red beet (*Beta vulgaris* L.) peel, which is generated as a by-product during red beet processing, possesses the potential to be utilized as a functional ingredient in food formulations due to the high-value components it contains. Red beet peel is remarkably rich in betalain pigments, which impart the characteristic red-violet color to the plant and exhibit potent antioxidant, antimicrobial, and radical scavenging properties. Furthermore, it possesses a rich profile in terms of phenolic compounds, dietary fiber, and essential minerals (1, 3, 4). These functional properties enable red beet peel to be utilized as a natural preservative and colorant that not only enhances the nutritional value of food products but can also extend their shelf life by suppressing lipid oxidation.

Meat and meat products, especially products like meatballs and minced meat, are quite susceptible to oxidation during processing and storage. Oxidation leads to off-odors, off-flavors, color deterioration, and nutritional value losses in the product (5, 6). Although synthetic antioxidants are employed to prevent these adverse effects, consumers' growing demand for natural and clean-label foods has directed researchers toward natural antioxidant sources (7, 8). In this context, incorporating red beet peel into meatball formulations presents an innovative approach to enhancing the oxidative stability and mineral content of the product.

However, the frequency of consumption and popularity of meat products such as meatballs necessitate the investigation of effects of cooking methods on product quality and safety. Frying is a common cooking method that takes place at high temperatures and imparts desirable sensory attributes to meatballs (9, 10). Nonetheless, the frying process performed at high temperatures and in various oil media can induce thermal oxidation in foods and the formation of undesirable compounds, such as acrylamide, a potential carcinogen. Acrylamide is formed primarily through the Maillard reaction between carbohydrates and amino acids (particularly asparagine) at high temperatures. It is well-established that numerous parameters—such as pH, temperature, cooking temperature, and duration—influence acrylamide formation in food products. Indeed, posing a significant risk to human and animal health, acrylamide has been classified as a ‘probable human carcinogen' by the International Agency for Research on Cancer (11, 12). Recent literature studies suggest that plant-derived materials may exert a mitigating effect on acrylamide formation during thermal processing (2).

Although the utilization of red beet and its components in meat products has been investigated in the literature regarding general qualitative attributes (13–17), studies addressing the combined effects of different frying oils on the quality and, most importantly, the acrylamide formation of these enriched products are quite limited. The objective of this study was to investigate the alterations occurring during the frying process of meatballs functionally and nutritionally enriched with red beet peel powder (RBPP), using different oils. Within the scope of the study, the effects of different oil types on various quality parameters of meatballs (pH, moisture content, cooking loss, color, lipid oxidation and texture) and their acrylamide content—a critical parameter regarding food safety—were comprehensively evaluated. The findings obtained are aimed at contributing both to the sustainable management of food waste and to the processing technologies of healthier and safer meat products.

## 2 Material and methods

### 2.1 Raw material

In the study, the M. *gluteus medius* muscle and intramuscular fat obtained from beef carcasses were utilized as materials. The M. *gluteus medius* muscles were dissected and separated from the carcasses aged for 24 h post-mortem. The beef samples, transported to the laboratory under a cold chain, were trimmed of visible external fat and connective tissues, and subsequently homogenized for use in the applications. Red beet (*Beta vulgaris* L.), used as a plant additive and functional raw material in the study, was obtained fresh from a local market. The preparation of the RBPP was carried out through the following stages: washing, sorting, peeling, and drying. Beet peels were manually separated to remove any unsuitable parts before processing began. Drying: The obtained beet peels were dried in an oven adjusted to 50°C until reaching a constant weight, in order to minimize the adverse effects of thermal processing on bioactive components. The dried peels were ground into a fine powder using a laboratory-scale grinder. It was then passed through a sieve with a pore diameter of 500 μm. The prepared RBPP was stored in hermetically sealed, dark-colored glass jars to protect it against moisture and light. The pH of the meat used was determined to be 5.59 ± 0.01, while its moisture content was in the range of 72.23 ± 0.27%.

### 2.2 Meatball production and cooking process

For the preparation of experimental samples, the M. *gluteus medius* muscles, which were trimmed of connective tissue and external fat, were homogenized by passing through a meat grinder. The resulting meatball batter containing 20% fat was divided into three equal portions. RBPP at the specified concentrations (0%, 0.5%, and 1%) were added to the meat samples and mixed thoroughly to ensure a homogenous distribution of the additive within the meat matrix.

The shaped beef meatball samples (approximately 60 g) were subjected to a stainless steel pot cooking process to investigate effects of different cooking media (oil types) and RBPP on the samples. The cooking process was performed in a standard stainless steel pot with homogenous heat distribution. For each cooking period, 400 mL of the respective cooking oil (sunflower oil, corn oil, and hazelnut oil) was added to the steel pot. The oils were evaluated separately rather than as a mixture to enable comparison of their individual effects under identical cooking conditions. To ensure standardized cooking conditions, the cooking temperature was set at 200 C and the cooking time remained constant in all experiments. Regarding the cooking duration, the meatball samples were placed in the pan once the oil temperature reached 200 C and were cooked for a total of 4 min (flipped to ensure both sides were homogenously exposed to heat). After the cooking process was completed, the meatballs were removed from the pan, kept on a wire strainer for a short period to remove excess surface oil, and subsequently cooled to room temperature prior to analyses.

### 2.3 Analyses

#### 2.3.1 pH assay

The pH values of the raw and cooked meatball samples were determined according to Gökalp et al. (18). The pH value of the obtained homogenate was measured at room temperature by immersing a digital pH meter (FiveEasy Plus FP20, Mettler Toledo), which had been calibrated with standard buffer solutions prior to measurement. The measurements were performed in triplicate for each sample, and the arithmetic mean of the results was recorded.

#### 2.3.2 Moisture assay

The moisture content of the raw and cooked meatball samples were determined according to Gökalp et al. (18). For this purpose, approximately 5 g of meatball sample from each experimental group was weighed homogenously and subjected to a drying process until reaching a constant weight in a laboratory dry oven (WiseVen, Fuzzy Control System) adjusted to a temperature of 105 ± 2°C, which had been previously brought to a constant weight. The percentage (%) moisture contents were calculated based on the weight differences of the samples before and after drying.

#### 2.3.3 Cooking loss assay

The cooking loss values of the meatball samples were calculated based on the difference between the precise weighings performed before and after the cooking process (19).


$$\begin{array}{l} \text{Cooking Loss }\left(\text{\%}\right)=\frac{{\text{W}}_{1-}{\text{W}}_{2}}{{\text{W}}_{1}}\times 100 \end{array}$$

Where;

W_1_ = weight of sample before cooking (g)

W_2_ = weight of sample after cooking (g).

#### 2.3.4 Color

The color properties of meatball samples were determined on both raw and cooked samples using a colorimeter (3nh NR100, China) according to the standard system defined by the International Commission on Illumination (CIE). Following the measurements performed on at least three different randomly selected points from the inner and outer surfaces of the samples; L^*^, a^*^, and b^*^ values were recorded. The final color data of the meatballs for each experimental group were calculated by taking the arithmetic mean of these measurements.

#### 2.3.5 Texture asssay

The textural properties of the cooked beef meatball samples were determined according to the dual-compression Texture Profile Analysis (TPA) method using a texture analyzer (TA.XT Plus, Stable Micro Systems, Godalming, UK) on standard cylindrical specimen cut from the core regions of the samples cooled to room temperature (2, 8). A 36-mm diameter cylindrical probe (P/36, 5 kg load cell) was utilized in the measurements; the pre-test, test, and post-test speeds of the device were adjusted to 2.0 mm/s, 1.0 mm/s, and 1.0 mm/s, respectively, and the meatballs were compressed twice consecutively to 35% of their original height. Based on the force-time curves obtained from this process, the hardness, springiness, chewiness, and cohesiveness parameters of the samples were automatically calculated and recorded via the device software.

#### 2.3.6 Acrylamide assay

The acrylamide content in the meatball samples was determined according to the method described in the literature Baharinikoo et al. (20) and Savaş et al. (2). Accordingly, 0.1 mL of Carrez I, 0.1 mL of Carrez II, and 9.8 mL of acetic acid (0.2 mM) were added to 1 g of homogenized sample, and the mixture was shaken for 2 min. Following the extraction process, the samples were centrifuged at 5,000 rpm for 10 min. The supernatant was filtered through a 0.45-μm syringe filter and injected into the HPLC system. A THERMO-Acclaim™ 120-C18 (3 μm, 120 Å, 4.6 × 150 mm; Waltham, MA, USA) column was utilized for chromatographic separation; the flow rate was set to 1 mL/min, and the column temperature was maintained at 36 °C. The analyses were detected within a wavelength range of 206–400 nm. Acrylamide standard solutions were prepared at 0.25–10 ng/ul, and the calibration curve showed good linearity (R^2^ = 0.99). All measurements were performed in two independent replicates. The mobile phase consisted of solvent A (water containing 0.1% phosphoric acid) and solvent B (methanol). The retention time of acrylamide was determined as 3.053 min. The limit of detection (LOD) and limit of quantification (LOQ) values for the calibration curve created with acrylamide standards were determined as 12.99 μg/kg and 39.37 μg/kg, respectively.

#### 2.3.7 Lipid oxidation assay

The degree of lipid oxidation in beef meatball samples was evaluated using the thiobarbituric acid reactive substances (TBARS) assay, adapted from the method described by Kilic and Richards (21) with slight modifications. A UV–Vis spectrophotometer (UVmini-1240, Shimadzu, Kyoto, Japan) was utilized to measure the absorbance at a wavelength of 532 nm.

#### 2.3.8 Statistical analysis

The data obtained from the study were analyzed according to a two-factor full factorial experimental design (3 oil types × 3 concentrations). All experimental treatments were performed using two independent replicates (*n* = 2). Two-Way Analysis of Variance (Two-Way ANOVA) was applied to determine the effects of oil types, concentration levels, and the interaction of these two factors (oil × concentration) on the dependent variables. In cases where the *p*-value was found to be significant (*p* < 0.05) as a result of ANOVA, Duncan's multiple comparison test was used to compare the group means of the main effects and interaction variances. Principal Component Analysis (PCA) was performed to holistically evaluate the effects of different oil types and concentration applications on the general parametric profile, to visualize the similarities/differences among the samples, and to determine the relationships between variables. Variance analysis and Duncan's tests were conducted using the SPSS 25.00 software, while PCA and the plotting of graphics were performed using the SIMCA 14.1 (UMETRICS, Umea, Sweden) software.

## 3 Results and discussion

The pH values of the meatball samples are summarized in Table 1. The concentration of RBPP was determined to have a statistically significant impact on the pH values of meatball samples (*p* < 0.05). The pH values of the samples ranged between 6.22 and 6.25. While the control group exhibited the lowest pH value (6.22), the addition of 0.5% concentration significantly increased the pH value, reaching the highest level (6.25). However, increasing the concentration to 1% caused a slight decrease in the pH value (6.24). The effect of oil type on the pH value of meatballs was found to be highly significant (*p* < 0.01). The highest pH value was detected in samples containing sunflower oil (6.29), followed by hazelnut oil (6.25) and corn oil (6.16), respectively. This difference may be associated with differences in the physicochemical characteristics of the oils; however, the specific mechanisms underlying this effect require further investigation. The binary interaction between concentration and oil type (C × TO) was found to be statistically significant on the pH value (*p* < 0.01). This indicates that the effect of additive concentration on pH varies depending on the type of oil utilized. The pH values obtained in the present study (6.22–6.25) are within the range reported by Lu et al. (22), who observed pH values between 5.97 and 6.28 in meatballs formulated with different spices. However, our values were consistently higher than those reported by Savaş et al. (2) (5.95–6.03), which may be attributed to differences in the type of plant-based ingredient incorporated, formulation composition, and processing conditions. The relatively small changes in pH observed with RBPP addition indicate that this ingredient can be incorporated into meatball formulations without markedly altering the product's acid–base balance.

**Table 1:** Effects of frying with different oil types and RBPP incorporation levels on pH value, moisture content, and cooking loss of meatballs.

Concetrations (%)	pH	Moisture content (%)	Cooking loss (%)
0 (Control)	6.22 ± 0.05b	55.08 ± 1.65a	26.11 ± 1.72
0.5	6.25 ± 0.10a	50.51 ± 4.73b	26.66 ± 7.11
1	6.24 ± 0.11ab	54.14 ± 4.20ab	24.66 ± 8.91
Sig.	^*^	^*^	ns
Type of oil			
Sunflower oil	6.29 ± 0.11a	50.45 ± 4.77b	31.27 ± 5.31a
Corn oil	6.16 ± 0.06c	55.45 ± 2.11a	20.27 ± 5.23c
Hazelnut oil	6.25 ± 0.02b	53.83 ± 3.73ab	25.88 ± 2.71b
Sig.	^**^	^*^	^**^
C × TO			
Sig.	^**^	ns	^*^

^*^, significant (*p* < 0.05); ^**^, very significant (*p* < 0.01); ns, insignificant (*p* > 0.05); Sig., significance level. Means with different letters (a–c) in the same column are significantly different. Different concentrations (C)—0%, 0.5%, and 1% and type of oil (TO)—sunflower oil, corn oil, hazelnut oil.

It was determined that both concentration and oil type had a statistically significant effect on the moisture content (*p* < 0.05). When concentration levels were examined, the control group (0%) exhibited the highest moisture value at 55.08%. At a concentration of 0.5%, this value experienced a sharp decline, dropping to the lowest level (50.51%). However, at a 1% concentration, the moisture content increased again to 54.14%, placing it in a statistically similar group to the control. In terms of oil types, the highest moisture content was found in samples cooked in corn oil at 55.45%, followed by hazelnut oil at 53.83%; the lowest moisture content was found in samples cooked in sunflower oil at 50.45%. The moisture content observed in the present study (50.51–55.08%) was within the range reported for similar meat products but differed from some previous studies. While our results were close to those reported by Korkmaz and Oz (23), they were lower than those reported by Elbir et al. (24) and higher than those reported by Lu et al. (22). These differences may be attributed to variations in meat formulation, the physicochemical properties of the incorporated plant materials, and differences in processing conditions. In particular, ingredients with higher dietary fiber and gel-forming capacities, such as chia, are expected to retain more water than red beet peel powder, whereas differences in meat type and formulation may account for the lower moisture values reported by Lu et al. (22).

According to the statistical analysis results, the effect of RBPP concentration on cooking loss was found to be insignificant, whereas the effect of oil type was determined to be highly significant (*p* < 0.01). When the concentration factor was examined individually; the control (0%), 0.5%, and 1% levels exhibited cooking loss values of 26.11%, 26.66%, and 24.66%, respectively. This indicates that the increase in the concentration of the ingredient used could not radically alter the overall water and oil holding capacity of the product on its own. In contrast, highly distinct differences were observed among oil types; the highest cooking loss was detected in samples containing sunflower oil with 31.27%, while the lowest loss was determined in samples containing corn oil with 20.27%, and hazelnut oil ranked between these two groups with 25.88%. These cooking loss values, obtained from different meat products and with various additives in the literature, show a general consensus with the results obtained in the current study. The cooking loss values obtained in the present study (24.66–26.66%) were comparable to those reported by Lu et al. (22) (25.59–31.28%) but were lower than those reported by Pietrasik et al. (25) and Elbir et al. (24). In contrast, the values reported by Korkmaz and Oz (23) covered a much wider range that also included the results of the present study. Such differences among studies are expected because cooking loss is influenced by several factors, including meat composition, fat level, processing conditions, and the functional properties of the incorporated ingredients.

Color is one of the most important quality attributes influencing consumer preference (26), and the composition of meat products directly affects this parameter (27). In the present study, the concentration of RBPP added to the formulation and the type of oil used did not have a statistically significant effect on the L^*^, a^*^, b^*^, C^*^, and h° values of the samples (*p* > 0.05). The L^*^ values of the samples ranged from 22.18 to 27.58, a^*^ values from 6.82 to 10.57, b^*^ values from 13.83 to 16.41, chroma (C^*^) values from 17.47 to 18.30, and hue angle (h°) values from 53.02 to 67.81 (Table 2).

**Table 2:** Effects of RBPP incorporation levels and frying oil types on L^*^, a^*^, b^*^, chroma, and hue values of meatballs.

Concetrations (%)	L^*^	a^*^	b^*^	C^*^	h°
0 (Control)	27.58 ± 3.48	10.57 ± 2.54	13.83 ± 1.16	17.47 ± 2.30	53.02 ± 4.97
0.5	22.18 ± 5.79	6.82 ± 4.66	16.41 ± 2.58	18.30 ± 2.42	67.81 ± 14.50
1	23.02 ± 2.65	7.21 ± 3.81	15.84 ± 2.83	17.77 ± 2.66	65.65 ± 12.64
Sig.	ns	ns	ns	ns	ns
Oil					
Sunflower oil	22.83 ± 5.54	6.11 ± 3.59	14.99 ± 2.19	16.54 ± 1.96	67.81 ± 12.69
Corn oil	24.79 ± 3.65	9.23 ± 3.90	15.96 ± 2.90	18.81 ± 2.66	60.08 ± 12.33
Hazelnut oil	25.16 ± 5.01	9.25 ± 4.12	15.13 ± 2.56	18.18 ± 2.09	58.58 ± 13.25
Sig.	**ns**	**ns**	**ns**	**ns**	**ns**
C × TO					
Sig.	ns	ns	ns	ns	ns

^*^, significant (*p* < 0.05); ^**^, very significant (*p* < 0.01); ns, insignificant (*p* > 0.05); Sig., significance level. Means with different letters (a–c) in the same column are significantly different. Different concentrations (C)—0%, 0.5%, and 1% and type of oil (TO)—sunflower oil, corn oil, hazelnut oil.

Although the differences were not statistically significant, an increase in the proportion of RBPP resulted in a numerical tendency toward lower L^*^ and a^*^ values and higher b^*^, C^*^, and h° values compared with the control group. A review of the literature showed that Akcan et al. (27) reported L^*^, a^*^, and b^*^ values ranging from 39.97 to 43.37, 2.06 to 2.27, and 2.59 to 3.49, respectively, on day 0 of cooked meatballs subjected to different processing treatments. Liu et al. (8), in their study on beef meatballs, reported L^*^, a^*^, and b^*^ values ranging from 55.07 to 58.11, 6.37 to 7.10, and 13.00 to 14.56, respectively. Similarly, Qing et al. (26) reported significant differences among samples treated with different concentrations of tea polyphenols, with L^*^, a^*^, and b^*^ values ranging from 36.40 to 46.55, 11.65 to 13.61, and 11.87 to 18.31, respectively, in ultrasound-treated boiled beef meatballs. Savaş et al. (2), in a beef meatball study, reported that the addition of sour cherry seed powder resulted in L^*^, a^*^, b^*^, C^*^, and h° values ranging from 34.40 to 36.62, 7.44 to 8.59, 6.84 to 8.93, 10.16 to 12.41, and 41.35 to 46.17, respectively.

When compared with the literature, the L^*^ values obtained in the present study were generally lower, indicating a darker product appearance. On the other hand, the redness (a^*^) and yellowness (b^*^) values were found to be comparable to or higher than those reported in some previous studies, which may be attributed to the natural pigment composition of the RBPP, the cooking process, and vegetable oils used in the formulation.

The textural properties of meatballs containing different concentrations of RBPP are summarized in Table 3. Both the concentration of RBPP and the type of oil had a significant effect on the texture characteristics of the samples. The hardness values of the meatball samples ranged from 38.86 to 46.60 N. An increase in hardness was observed with the addition of RBPP, with the highest hardness value recorded in meatballs containing 1% RBPP. This increase may be attributed to the dietary fiber content of beet peel powder, which enhances water-holding capacity and contributes to the formation of a more compact and rigid matrix structure (28). Regarding the effect of oil type, the highest hardness value was observed in samples formulated with sunflower oil, whereas the lowest value was determined in samples containing hazelnut oil. Akcan et al. (27) reported hardness values ranging from 27.20 to 30.55 N in cooked meatballs subjected to different processing treatments and found no significant differences among the samples. In contrast, Liu et al. (8) reported hardness values ranging from 13.02 to 15.58 N in beef meatballs and observed statistically significant differences compared with the control group. Furthermore, the incorporation of RBPP increased the hardness values of the samples. Similarly, Savaş et al. (2) reported hardness values ranging from 3.17 to 4.98 N in beef meatballs and indicated significant differences among the treatment groups.

**Table 3:** Effects of RBPP incorporation levels and frying oil types on the texture profile properties of meatballs.

Concentrations (%)	Hardness (N)	Springiness (mm)	Cohesiveness	Gumminess (N)	Chewiness (N·mm)	Resillience
0 (Control)	38.86 ± 8.17b	0.85 ± 0.05	0.67 ± 0.10	26.52 ± 8.15	23.03 ± 8.62	0.34 ± 0.06
0.5	45.42 ± 8.25ab	0.87 ± 0.07	0.62 ± 0.10	28.43 ± 8.06	25.19 ± 9.18	0.34 ± 0.01
1	46.60 ± 9.65a	0.84 ± 0.02	0.62 ± 0.09	29.53 ± 9.18	25.07 ± 7.95	0.33 ± 0.01
Sig.	^*^	ns	ns	ns	ns	ns
Type of oil						
Sunflower oil	50.45 ± 8.48a	0.90 ± 0.06a	0.71 ± 0.08	35.82 ± 6.45a	32.66 ± 7.59a	0.37 ± 0.05a
Corn oil	46.03 ± 3.09a	0.82 ± 0.02b	0.61 ± 0.09	27.92 ± 5.10b	23.08 ± 3.86b	0.32 ± 0.02b
Hazelnut oil	34.40 ± 4.81b	0.84 ± 0.02ab	0.60 ± 0.08	20.72 ± 4.11b	17.55 ± 3.55b	0.33 ± 0.02b
Sig.	^**^	^*^	**ns**	^**^	^**^	^*^
C × TO						
Sig.	ns	ns	ns	ns	ns	ns

^*^, significant (*p* < 0.05); ^**^, very significant (*p* < 0.01); ns, insignificant (*p* > 0.05); Sig., significance level. Means with different letters (a–c) in the same column are significantly different. Different concentrations (C)—0%, 0.5%, and 1% and type of oil (TO)—sunflower oil, corn oil, hazelnut oil. N, Newton; mm, millimeter.

The addition of RBPP did not have a statistically significant effect on the springiness values of the samples. The springiness values of the meatball samples ranged from 0.84 to 0.87 mm. Although no significant differences were observed, variations in springiness were noted with the incorporation of RBPP, and the highest springiness value was recorded in meatballs containing 0.5% RBPP. Savaş et al. (2) reported that the springiness values of beef meatballs ranged from 0.95 to 0.99 mm and that significant differences existed among the samples. On the other hand, the type of oil used had a significant effect on the springiness values of the meatball samples. Akcan et al. (27) reported springiness values ranging from 4.28 to 4.46 mm in cooked meatballs subjected to different processing treatments and observed significant differences among the treatment groups. Similarly, Liu et al. (8) reported that the springiness values of meatball samples ranged from 2.78 to 3.10 and increased significantly compared with the control group. These differences can be attributed to variations in meat formulation, fat source, processing conditions, fiber type and concentration, cooking methods, and texture profile analysis. Overall, the results show that RBPP has only a limited effect on elasticity at the tested levels, but the choice of fat source has a greater influence on the elastic properties of the meatball samples.

Neither the concentration of RBPP nor the type of oil had a statistically significant effect on the cohesiveness values of the meatball samples. The cohesiveness values ranged from 0.62 to 0.67. A decreasing trend in cohesiveness was observed with increasing levels of RBPP incorporation. Regarding the effect of oil type, the highest cohesiveness value was recorded in samples formulated with sunflower oil. Akcan et al. (27) reported cohesiveness values ranging from 0.63 to 0.74 in cooked meatballs subjected to different processing treatments and found no significant differences among the samples. Similarly, Liu et al. (8) reported cohesiveness values ranging from 0.48 to 0.50 in meatball samples and indicated that the differences among treatments were not statistically significant. Likewise, Savaş et al. (2) reported cohesiveness values ranging from 0.28 to 0.39 in beef meatballs and observed no significant differences among the samples. The cohesiveness values obtained in the present study were generally higher than those reported by Liu et al. (8) and Savaş et al. (2), while being comparable to the values reported by Akcan et al. (27). These differences may be associated with variations in formulation composition, processing conditions, and the functional properties of the ingredients used.

The concentration of RBPP did not have a statistically significant effect on the gumminess values of the meatball samples. The gumminess values ranged from 26.52 to 29.53 N. Although the differences were not statistically significant, gumminess values increased in samples containing RBPP compared with the control group. On the other hand, the type of oil significantly affected the gumminess values, with the highest gumminess being observed in samples formulated with sunflower oil. Akcan et al. (27) reported gumminess values ranging from 18.73 to 21.48 N in cooked meatballs subjected to different processing treatments and found no significant differences among the samples. Similarly, Liu et al. (8) reported gumminess values ranging from 6.25 to 7.69 in meatball samples and observed an increase in gumminess compared with the control group. Savaş et al. (2) reported gumminess values ranging from 1.22 to 1.41 N in beef meatballs and indicated that the differences among samples were not statistically significant.

The concentration of RBPP also did not significantly affect the chewiness values of the meatball samples. Chewiness values ranged from 23.03 to 25.19 N·mm. Similar to gumminess, chewiness values increased in samples containing RBPP compared with the control group. However, the type of oil had a significant effect on chewiness, and the highest chewiness value was determined in samples formulated with sunflower oil. Akcan et al. (27) reported chewiness values ranging from 79.23 to 94.45 mJ in cooked meatballs subjected to different processing treatments and observed significant differences among the samples. Liu et al. (8) reported chewiness values ranging from 17.32 to 23.62 and noted that chewiness increased compared with the control group. Likewise, Savaş et al. (2) reported chewiness values ranging from 1.19 to 1.39 N·mm in beef meatballs and found no significant differences among the treatment groups. Compared with the findings reported in the literature, the gumminess and chewiness values obtained in the present study were generally higher than those reported by Liu et al. (8) and Savaş et al. (2), while showing partial agreement with the values reported by Akcan et al. (27). The observed increases in gumminess and chewiness may be associated with the dietary fiber-rich structure of RBPP, which could strengthen the meat matrix through enhanced water retention and improved structural integrity.

The concentration of RBPP also did not have a statistically significant effect on the resilience values of the meatball samples. Resilience values ranged from 0.33 to 0.34. In contrast, oil type significantly affected resilience, with the highest resilience value observed in samples formulated with sunflower oil. Overall, the increases observed in hardness, umminess, chewiness, and resilience may be associated with the structural contribution of RBPP and the functional properties of the oils used in the formulations. The dietary fiber present in beet peel powder may enhance water retention and matrix integrity, thereby contributing to improved textural characteristics of the meatball samples.

The acrylamide (AA) contents of meatballs cooked with different concentrations of RBPP and different vegetable oils are presented in Table 4. According to the statistical analysis, the addition of red beet peel powder RBPP had a significant effect on the acrylamide content of the meatball samples (*p* < 0.05). The acrylamide (AA) contents of meatballs prepared with different concentrations of RBPP and different vegetable oils are presented in Table 4. According to the statistical analysis, the addition of red beet peel powder RBPP had a significant effect on the acrylamide content of the meatball samples (*p* < 0.05). The mean acrylamide (AA) contents of the meatball samples were 63.78, 72.35, and 64.53 μg/kg for the control, 0.5% RBPP, and 1% RBPP treatments, respectively. Compared with the control group, supplementation with 0.5% red beet peel powder (RBPP) significantly increased acrylamide formation (*p* < 0.05), corresponding to an increase of approximately 13.44%. In contrast, increasing the RBPP concentration to 1% did not result in a significant difference compared with the control group (*p* > 0.05). These findings indicate that the effect of RBPP on acrylamide formation was concentration-dependent but not linear. Acrylamide is primarily generated through the Maillard reaction between free asparagine and reducing sugars during thermal processing. Red beet contains free amino acids, including asparagine, which is known as a precursor to reducing sugars and acrylamide formation. Adding red beet peel to meat products may increase the potential for acrylamide formation during high-temperature cooking; however, acrylamide formation depends on the concentration of these precursors, processing conditions, and product formulation. Choińska et al. (29) reported that asparagine is one of the predominant free amino acids in red beet, supporting the hypothesis that RBPP supplementation can increase the availability of acrylamide precursors. However, the absence of a further increase at the 1% supplementation level suggests that additional mechanisms may influence acrylamide formation. The type of frying oil had no significant effect on acrylamide content (*p* > 0.05). Although numerical differences were observed among the oils, the mean acrylamide contents were 66.61, 68.37, and 65.69 μg/kg for sunf lower oil, corn oil, and hazelnut oil, respectively. Hazelnut oil produced the lowest numerical acrylamide value; however, these differences were not statistically significant, indicating that the formation of acrylamide was mainly influenced by RBPP supplementation rather than by the frying oil under the experimental conditions employed. The acrylamide concentrations obtained in the present study were generally within the range reported for thermally processed meat products. Demirhan et al. (30) reported acrylamide levels ranging from 24.6 to 71.3 μg/kg in different meat products, whereas Sun et al. (31) found substantially higher concentrations (251.57–284.47 μg/kg) in beef, pork, and chicken meatballs. More recently, Savaş et al. (2) reported acrylamide contents between 51.44 and 59.35 μg/kg in meatball samples and observed significant differences among formulations. Therefore, the acrylamide levels determined in the present study were considerably lower than those reported by Sun et al. (31), slightly higher than those reported by Savaş et al. (2), and generally comparable with the upper range reported by Demirhan et al. (30). This the results demonstrate that moderate RBPP supplementation (0.5%) promoted acrylamide formation, whereas increasing the supplementation level to 1% did not further enhance acrylamide production. Furthermore, frying oil type did not significantly influence acrylamide formation. The absence of a further increase at the 1% supplementation level suggests that acrylamide formation may be influenced by multiple factors beyond the concentration of RBPP alone.

**Table 4:** Acrylamide (AA) and TBARS contents of meatballs prepared with different RBPP incorporation levels and fried in different oil types.

Concentrations (%)	AA (μg/kg)	TBARS (mg MDA/kg)
0 (Control)	63.78 ± 7.36a	1.49 ± 0.43b
0.5	72.35 ± 6.94b	1.45 ± 0.17b
1	64.53 ± 5.21a	1.88 ± 0.13a
Sig.	^*^/	^*^
Type of oil		
Sunflower oil	66.61 ± 10.55	1.59 ± 0.43
Corn oil	68.37 ± 6.72	1.68 ± 0.24
Hazelnut oil	65.69 ± 4.76	1.55 ± 0.33
Sig.	**ns**	**ns**
C × TO		
Sig.	**ns**	**ns**

^*^, significant (*p* < 0.05); ^**^, very significant (*p* < 0.01); ns, insignificant (*p* > 0.05); Sig., significance level. Means with different letters (a–b) in the same column are significantly different. Different concentrations (C)—0%, 0.5%, and 1% and type of oil (TO)—sunflower oil, corn oil, hazelnut oil.

Figure 1 shows a graphical representation of acrylamide using Sankey visualization. The presented Sankey diagram quantitatively demonstrates the overall effects of different oils and RBPP ratios (0%, 0.5%, and 1%) on acrylamide. Examination of the total flows reveals that the highest contributions originate from the sunflower oil (213.17 μg/kg) and corn oil (205.12 μg/kg) conditions, whereas hazelnut oil (183.75 μg/kg) provides a comparatively limited contribution. From the perspective of red beet peel powder RBPP ratios, the 0.5% incorporation level (217.07 μg/kg) exhibits the highest total flow, significantly enhancing acrylamide formation. In contrast, the control samples (0%; 191.35 μg/kg) display a lower and more dispersed production profile, while the 0.5% RBPP addition (217.07 μg/kg) results in a most high increase.

**Figure 1:**
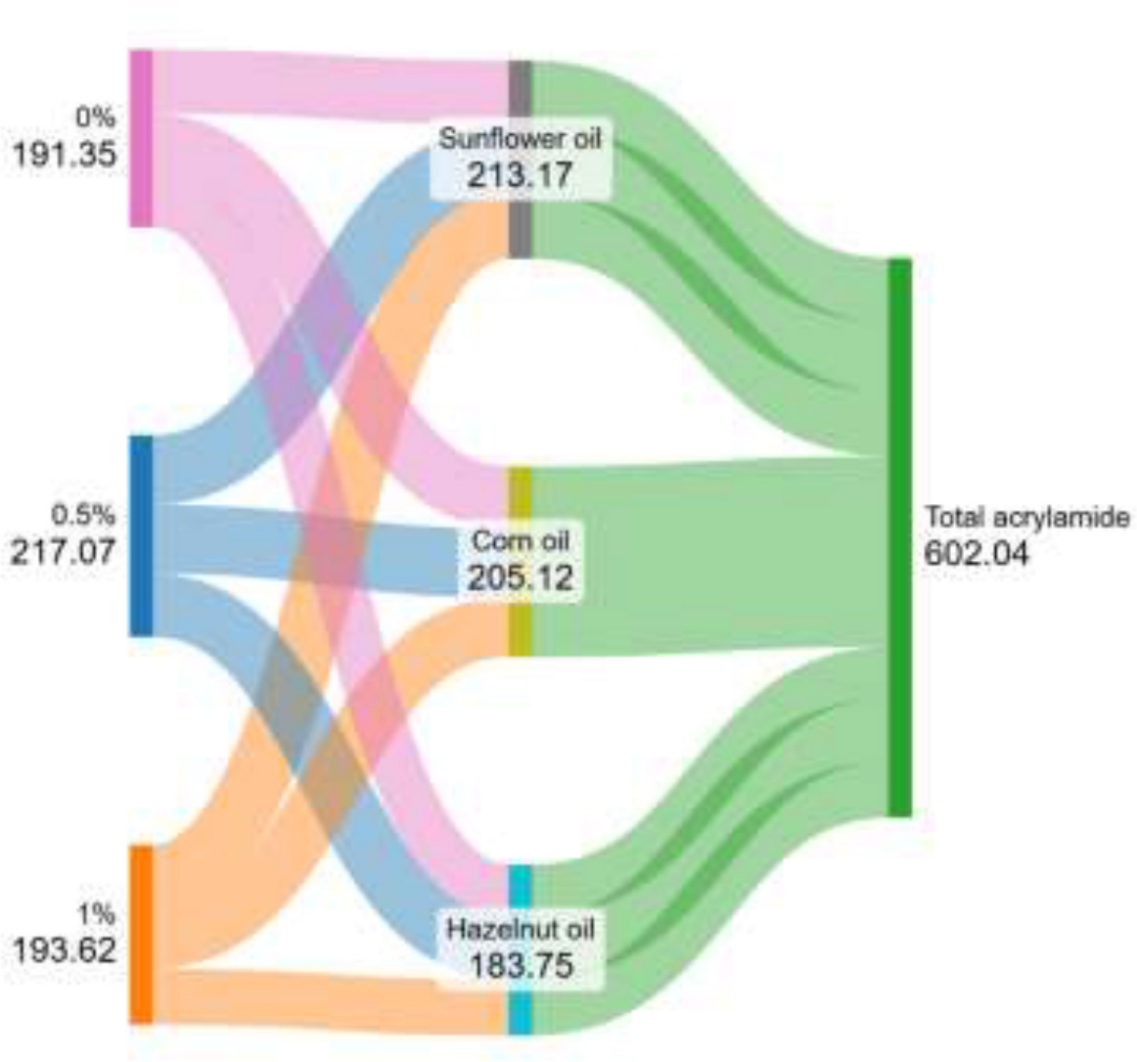
Grapical represantation of the acrylamide via Sankey visualization.

Data on TBARS content of meatball samples are summarized in Table 4. Lipid oxidation is one of the most fundamental chemical reactions leading to quality loss during the storage and processing of meat and meat products. TBARS analysis was applied to monitor the level of lipid oxidation in meat samples and the qualitative changes during the processing. While the proportion of RBPP significantly affected the meatball samples, the type of oil did not have a significant effect. In the study, the TBARS values of the meatball samples ranged from 1.45 to 1.88 mg MDA/kg (Table 4). Numerous studies have been conducted to control lipid oxidation in meat products through the use of natural antioxidants derived from plant sources. Almohaimeed et al. (32) reported that beef burgers incorporated with 3% and 6% fermented radish and beetroot mixture powder and subjected to cold storage for 14 days exhibited TBARS values ranging between 1.71 and 2.26 mg MDA/kg, with statistically significant differences observed among the treatments. Similarly, Savaş et al. (2) determined that TBARS values in meatball samples formulated with sour cherry seed powder ranged from 0.73 to 1.04 mg MDA/kg, reporting that the incorporation of sour cherry seed reduced lipid oxidation compared to the control group. The researchers attributed this effect to the high antioxidant activity of the sour cherry seed. On the other hand, Korkmaz and Öz (23) reported that TBARS values in meatball samples containing different proportions of dry bread crumbs ranged between 1.733 and 2.092 mg MDA/kg, and the differences among the groups were statistically significant. In a study conducted by Savaş et al. (33), it was determined that the TBARS value in meatball samples containing different levels of sumac was as high as 1.27 mg MDA/kg. The variations observed among the TBARS values reported in the literature can be attributed to several factors, such as the type and inclusion level of the plant material used, product formulation, fat content, storage conditions and duration, as well as the cooking temperature and time applied. Therefore, both product composition and processing conditions must be considered together when evaluating the level of lipid oxidation.

### 3.1 PCA analysis

PCA was applied to show the differences between meatballs with added RBPP by evaluating some chemical and physicochemical analyses, texture profile, acrylamide levels (Figures 2A–D). This Figures 2A–D presents a Principal Component Analysis (PCA) of RBPP –enriched meatball samples. The first two principal components (PC1: 44% and PC2: 20.6%) explain sta substantial portion of the total variance, allowing visualization of relationships between processing conditions and quality parameters. The chroma, a^*^, moisture, and L^*^ values are clustered in the right region, while other analyses are clustered in the left region (Figure 2B). Specifically, groups A-1 and A-0.5 were found to have the highest cooking loss, hardness, acrylamide and pH values (Figure 2C). Additionally, three different groups were formed, and these groups were shown in green, blue, and red (Figure 2D).

**Figure 2:**
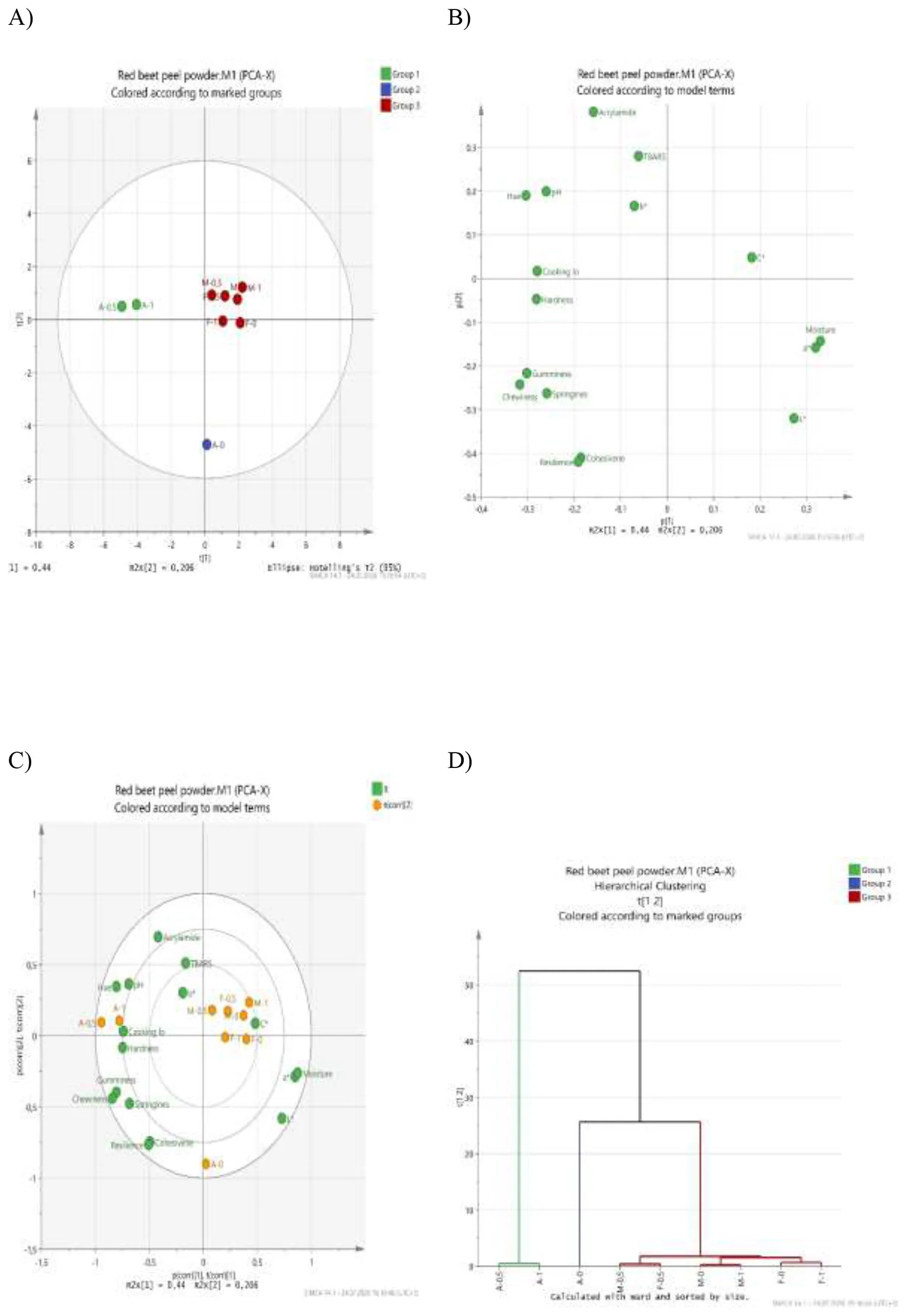
Score scatter plot **(A)**, loading scatter plot **(B)** biplot **(C)** and dendogram **(D)** of PCA analysis (PC1 vs. PC2) for the components in the meatball samples.

## 4 Conclusion

In the present study, it was demonstrated that the incorporation of red beet peel powder (RBPP) and the type of frying oil influenced the physicochemical, textural, and chemical properties of beef meatballs. Regarding the formulation, RBPP significantly affected pH, moisture content, TBARS values, and hardness, whereas no significant differences were observed in color parameters and most texture profile attributes. The highest acrylamide content was determined in the samples containing 0.5% RBPP. From a food quality and safety perspective, RBPP improved certain quality characteristics of beef meatballs; however, its effect on acrylamide formation varied depending on the formulation. Nevertheless, the findings indicate that further studies are required to optimize RBPP incorporation levels and processing conditions to maximize its functional benefits while minimizing the formation of undesirable compounds in meat products.

## Data Availability

The raw data supporting the conclusions of this article will be made available by the authors, without undue reservation.

## References

[1] Yeasmen N Bhuiyan MHR Gariepy Y Taherian AR Dumont MJ Ramaswamy H . Valorization of red beet peel through green extraction and carrier-driven microencapsulation for improved betalain stability. Food Chem. (2025) 498:147230. doi: 10.1016/j.foodchem.2025.147230 41308219

[2] Savaş A Kavrut E Engin T. Valorization of sour cherry seeds in beef meatballs: effect on quality, lipid oxidation, texture profile, acrylamide formation and antioxidant activity. Foods. (2026) 15:968. doi: 10.3390/foods15050968 41829241 PMC12985260

[3] Zin MM Bánvölgyi S. Emerging technology approach for extractability and stability of betalains from the peel of beetroot (*Beta vulgaris* L.). Biomass Convers Biorefin. (2023) 13:10759–69. doi: 10.1007/s13399-021-01975-z

[4] Nasim I Khalid N. Formulation and characterization of functional yogurt fortified with red beetroot peel polyphenols and color pigments encapsulated in water-in-oil-in-water emulsions. Appl Food Res. (2026) 6:101803. doi: 10.1016/j.afres.2026.101803

[5] Ungkusonmongkol P Wattanachant S. Fat composition and lipid oxidation in raw and cooked minced meat and skin of spent laying and broiler ducks. Appl Food Res. (2026) 6:101998. doi: 10.1016/j.afres.2026.101998

[6] Zhang Q Chen B Ma P Yi L Gu Y Wang S. Emerging insights into lipid oxidation in dry-cured meats: adverse effects on product quality and safety. J Future Foods. (2026). (In press). doi: 10.1016/j.jfutfo.2026.01.034

[7] Nishad J Koley TK Varghese E Kaur C. Synergistic effects of nutmeg and citrus peel extracts in imparting oxidative stability in meat balls. Food Res Int. (2018) 106:1026–36. doi: 10.1016/j.foodres.2018.01.075 29579894

[8] Liu S Chen X Tian Y Wang L Wu J Hu P. Enhancing beef meatballs quality with polysaccharides from Rosa roxburghii Tratt pomace: insights into physicochemical, textural, moisture distribution, sensory, and microstructural characteristics. Food Res Int. (2026) 231:118620. doi: 10.1016/j.foodres.2026.118620

[9] Kutlu N Meral R. A new strategy for reducing fat absorption and enhancing oxidative stability in fried chicken meatball using nanofiber coatings. Food Res Int. (2025) 221:117230. doi: 10.1016/j.foodres.2025.117230 41606843

[10] Wang Z Luo J Ng K Warner RD Stockmann R Fang Z. Effect of chitosan nanoparticle filled methylcellulose coatings on the polycyclic aromatic hydrocarbon levels of deep-fried meatballs. Food Control. (2025) 168:110946. doi: 10.1016/j.foodcont.2024.110946 40263845

[11] International Agency for Research on Cancer. Some industrial chemicals. Lyon: IARC Monographs on the Evaluation of Carcinogenicity Risks to Humans (1994). p. 60, 389–433.

[12] Hamad GM Abushaala MM Abushaala SM Ehmeza NT Ahmed EMH Elshaer SE . Acrylamide detection and reduction in meat products using organic acids, fruit extracts, and probiotics. Food Chem Toxicol. (2024) 192:114927. doi: 10.1016/j.fct.2024.114927 39134133

[13] Turp G Kazan H Ünübol H. Sosis üretiminde dogal renk maddesi ve antioksidan olarak kirmizi pancar tozu kullanimi. Celal Bayar Univ J Science. 12 (2016). doi: 10.18466/cbujos.76228

[14] Ayaseh A Alirezalu K Yaghoubi M Razmjouei Z Jafarzadeh S Marszałek K . Production of nitrite-free frankfurter-type sausages by combining ε-polylysine with beetroot extracts: an assessment of microbial, physicochemical, and sensory properties. Food Biosci. (2022) 49:101936. doi: 10.1016/j.fbio.2022.101936

[15] Sertdemirci Ö Gençcelep H. The investigation of the use red beet powder (Beta Vulgaris) as a natural colorant on color and antioxidant properties of heat treated sucuk. Black Sea J Agric. (2023) 6:304–13. doi: 10.47115/bsagriculture.1249523

[16] Tekin I Özcan K Ersus S. Optimization of ionic gelling encapsulation of red beet (Beta vulgaris L.) juice concentrate and stability of betalains. Biocatal Agric Biotechnol. (2023) 51:102774. doi: 10.1016/j.bcab.2023.102774

[17] del Amo-Mateos E Pérez R Merino A Lucas S García-Cubero MT Coca M. Rhamnogalacturonan–I pectin and derived oligosaccharides obtained from sugar beet pulp and discarded red beetroot: characterization and comparative study of their antioxidant and prebiotic properties. Food Hydrocolloids. (2024) 152:109955. doi: 10.1016/j.foodhyd.2024.109955

[18] Gökalp HY Kaya M Tülek Y Zorba Ö. Et ve ürünlerinde kalite kontrolü ve laboratuvar uygulama klavuzu. Erzurum: Atatürk Üniversitesi Yayinlari (2010).

[19] Savaş A. Farkli sicaklik ve baharat konsantrasyonlarinda airfryer kullanilarak pişirilen köftelerin bazi kalite özellikleri ile mineral içeriklerinin belirlenmesi. Turk J Agric Food Sci Technol. (2025) 13:2735–42. doi: 10.24925/turjaf.v13i9.2735-2742.7986

[20] Baharinikoo L Chaichi M Ganjali M. Detecting the quantity of acrylamide in potato chips utilizing CdTe surface functionalized quantum dots with fluorescence spectroscopy. Int J Peptide Resd Therap. (2020) 26:823–30. doi: 10.1007/s10989-019-09889-1

[21] Kilic B Richards MP. Lipid oxidation in poultry döner kebab: pro-oxidative and anti-oxidative factors. J Food Sci. (2003) 68:686–9. doi: 10.1111/j.1365-2621.2003.tb05732.x

[22] Lu F Kuhnle GK Cheng Q. The effect of common spices and meat type on the formation of heterocyclic amines and polycyclic aromatic hydrocarbons in deep-fried meatballs. Food Control. (2018) 92:399–411. doi: 10.1016/j.foodcont.2018.05.018

[23] Korkmaz A Oz F. Effect of the use of dry breadcrumb in meatball production on the formation of heterocyclic aromatic amines. Br Food J. (2020) 122:2105–19. doi: 10.1108/BFJ-04-2019-0256

[24] Elbir Z Ekiz E Aoudeh E Oz E Savaş A Brennan C . Enhancing effect of chia seeds on heterocyclic amine generation in meatball. Int J Food Sci Technol. (2023) 58:2560–72. doi: 10.1111/ijfs.16403

[25] Pietrasik Z Sigvaldson M Soladoye OP Gaudette NJ. Utilization of pea starch and fibre fractions for replacement of wheat crumb in beef burgers. Meat Sci. (2020) 161:107974. doi: 10.1016/j.meatsci.2019.107974 31704474

[26] Qing Z Meile W Yueling X Wen S Wengang J Linjie X. A machine learning approach to predict texture and sensory attributes of beef meatballs treated with ultrasound and tea polyphenols. LWT. (2026) 242:119152. doi: 10.1016/j.lwt.2026.119152

[27] Akcan T Önel E Ergezer H. Acorn (Quercus ithaburensis) Flour's effect on the physicochemical, textural, and sensory characteristics of raw and cooked beef meatballs. Int J Gastronomy Food Sci. (2024) 35:100887. doi: 10.1016/j.ijgfs.2024.100887

[28] Sabljak I Grubelić M Vlahović D Dukić J SamardŽija A ReŽek Jambrak A. Betaine and total dietary fiber recovery from red beetroot peels by means of ultrasound and pulsed electric fields. Clean Technol. (2025) 7:37. doi: 10.3390/cleantechnol7020037

[29] Choińska R Piasecka-Józwiak K Wozniak Ł Swider O Bartosiak E Bujak M . Starter culture-related changes in free amino acids, biogenic amines profile, and antioxidant properties of fermented red beetroot grown in Poland. Sci Rep. (2022) 12:20063. doi: 10.1038/s41598-022-24690-9 36414746 PMC9681880

[30] Demirhan B Demirhan BE Kara HES. Analysis of acrylamide in meat-based food products by using L-cysteine-capped Mn-doped ZnS quantum dots as a room temperature phosphorescent probe. Fresenius Environ Bull. (2019) 28:2599–607.

[31] Sun G Wang P Chen W Hu X Chen F Zhu Y. Simultaneous quantitation of acrylamide, 5-hydroxymethylfurfural, and 2-amino-1-methyl-6-phenylimidazo [4, 5-b] pyridine using UPLC-MS/MS. Food Chem. (2022) 375:131726. doi: 10.1016/j.foodchem.2021.131726 34954579

[32] Almohaimeed SA Al Juhaimi FY Babiker EE Mohamed Ahmed IA. Fermented radish and beetroot powder as natural sources of nitrite in beef burger. Foods. (2026) 15:1651. doi: 10.3390/foods15101651 42195854 PMC13206020

[33] Savaş A Ekiz E Elbir Z Savaş BD Proestos C Elobeid T . Advantageous effects of sumac usage in meatball preparation on various quality criteria and formation of heterocyclic aromatic amines. Separations. (2023) 10:29. doi: 10.3390/separations10010029

